# Quantitative in vivo and ex vivo confocal microscopy analysis of corneal cystine crystals in the *Ctns*^−/−^ knockout mouse

**Published:** 2011-08-17

**Authors:** Jennifer Simpson, Chyong Jy Nien, Kevin Flynn, Brian Jester, Stephanie Cherqui, James Jester

**Affiliations:** 1 Gavin Herbert Eye Institute, University of California, Irvine CA; 2 Scripps Research Institute, La Jolla CA

## Abstract

**Purpose:**

The purpose of this study was to assess the ability of quantitative in vivo confocal microscopy to characterize the natural history and detect changes in crystal volume in corneas from a novel animal model of cystinosis, the cystinosin (*Ctns^-/-^*) mouse.

**Methods:**

Two *Ctns*^−/−^ mice and one C57Bl/6 mouse were examined at each of the following time points: 2, 3, 5, 7, 10, 12, and 14 months of age. In vivo confocal microscopy scans were performed in 4 different regions of the cornea per eye. After, animals were sacrificed and cornea blocks evaluated for cell morphology using phalloidin and lymphocytic infiltration using CD45 antibodies by ex vivo confocal microscopy. Cystine crystal content in the cornea was measured by calculating the pixel intensity of the crystals divided by the stromal volume using Metamorph Image Processing Software.

**Results:**

Corneal crystals were identified in *Ctns*^−/−^ eyes beginning at 3 months of age and increased in density until 7–12 months, at which time animals begin to succumb to the disease and corneas become scarred and neovascularized. Older *Ctns*^−/−^ mice (7 months and older) showed the presence of cell infiltrates that stained positively for CD45 associated with progressive keratocyte disruption. Finally, at 12 months of age, decreased cell density and endothelial distortion were detected.

**Conclusions:**

Confocal microscopy identified corneal crystals starting at 3 month old *Ctns*^−/−^ eyes. Cystine crystals induce inflammatory and immune response with aging associated with loss of keratocyte and endothelial cells. These findings suggest that the *Ctns*^−/−^ mouse can be used as a model for developing and evaluating potential alternative therapies for corneal cystinosis.

## Introduction

Cystinosis is an autosomal recessive lysosomal storage disease that is characterized by the accumulation of cystine crystals in various tissues, including the kidney, brain and eye [1-4]. The disease has been linked to mutations in the cystinosin  (*CTNS*) gene (17p13) that codes for cystinosin, a trans-membrane protein responsible for the transport of the disulfide cystine amino acid out of the lysosome [5-9]. Different *CTNS* mutations are associated with a spectrum of clinical disease, with patients categorized into one of three severity groups based on their age at onset and symptoms [10]. The infantile onset of the most severe form of the disease, nephropathic cystinosis, results from the complete lack of cystine transport, with onset of renal failure by age 10 [11,12]. Corneal cystine crystals appear within the first 16 months of life, increasing linearly during the first decade, until they plateau in early adolescence [13-16]. The non-nephropathic forms of cystinosis (juvenile and ocular cystinosis) demonstrate some residual membrane transport function and are associated with later onset and more limited systemic manifestations. However, corneal crystals are also present in these less severe forms of the disease [17].

Oral administration of cysteamine (HS-CH_2_-CH_2_-NH_3_) or β-mercaptoethylamine has been the mainstay of cystinosis therapy since 1994, when CYSTAGON® (cysteamine bitartrate, Mylan Pharmaceuticals Inc., Canonsburg, PA) was approved by the USA FDA. A free thiol, cysteamine reacts with cystine to produce the single sulfide amino acid cysteine, plus a cysteine-cysteamine mixed disulfide that exits the lysosome via the lysine transporter. By circumventing the transporter defect, oral cysteamine has significantly improved overall prognosis [12,18-20]. However, no improvements in ocular manifestations of the disease have been demonstrated with systemic treatment, presumably due to the lack of bioavailability of the drug in the cornea [11,21-23].

Cysteamine can be applied directly to the cornea via eye drops and is effective in reducing corneal crystals. However, the hourly dosing regimen that this medication requires is impractical for effective compliance [24]. Progressive photophobia, recurrent corneal erosions, foreign body sensation and visual deterioration associated with increasing concentrations of corneal crystals have therefore become a major long-term burden for patients having all forms of cystinosis [14,25,26].

The low levels of intracorneal cysteamine achieved via topical application are thought to result from poor compliance, limited corneal penetration and drug instability [15,21,22,27-31]. Development of alternative treatments for corneal disease has been hampered by the need for costly and time consuming human clinical trials in this low prevalence disease [20,32,33]. Further, the lack of an objective, quantitative methodology to assess corneal treatment efficacy has forced studies to rely on subjective patient complaints and semi-quantitative slit-lamp biomicroscopy grading.

Recently, a cystinosis (*Ctns*^−/−^) knockout mouse has been shown to develop cystine crystals in multiple tissues, including the cornea [34,35]. The ocular findings in the *Ctns*^−/−^ mouse have been further characterized using slit lamp photography, assays of cystine levels in various ocular tissues, and standard histology. Cystine crystals were identified in the cornea as early as three months of age, while ocular tissue levels increased dramatically up to approximately 9–13 months. Most animals died between 13 and 20 months, with corneal vascularization identified histologically in older animals. Importantly, corneal cystine crystals were located within keratocytes throughout the stroma, but not in the epithelium, Bowman’s membrane, Descemet’s membrane or endothelium at any age [36].

The corneal findings in this murine knockout model appear to mimic disease progression in humans, where rapid linear increases in corneal crystals have been noted between birth and 6 years of age, with a plateau reached in the mid teenage years. Subsequent in vivo confocal microscopy studies in human nephropathic cystinosis patients 8–20 years old have demonstrated needle shaped hyperreflective bodies in the corneal epithelium and stroma of all patients [37-41].

In this study, we evaluated a quantitative method of confocal microscopy to characterize the natural history and localization of corneal crystal accumulation in the *Ctns*^−/−^ mouse model. This further development of the *Ctns*−/− mouse model may enable more rapid and cost-effective screening of potential alternative therapies for corneal cystinosis.

## Methods

### Mice

A total of seven C57Bl/6 (normal control) mice and 14 *Ctns*^−/−^ knockout mice were used in this study. Two *Ctns*^−/−^ mice and one C57Bl/6 mouse were examined at each the following periods: 2, 3, 5, 7, 10, 12 and 14 months of age. This timeframe was developed from initial studies that showed most animals surviving to about 12 months, by which time severe ocular changes, including phthisis bulbi began to be noted.

At each time point, the selected animals were anesthetized with intraperitoneal injections of ketamine HCl (100 mg/Kg bodyweight; Bioniche Pharma, Lake Forest, IL) and xylazine (20 mg/Kg bodyweight; Lloyd Laboratories, Shenandoah, IO). Clinical abnormalities were then detected using slit lamp biomicroscopy and in vivo confocal microscopy (CM) to assess the presence and location of cystine crystals. Animals were then sacrificed by cervical dislocation under anesthesia, after which ex vivo evaluations were performed. All procedures were approved by the UCI IACUC and conducted in accordance with ARVO Statement for the Use of Animals in Ophthalmic and Vision Research.

### Slit lamp photography

Selected eyes were photographed using a Nikon D200 digital camera (Nikon Corporation, Tokyo, Japan) attached to a Nikon NS-1 slit lamp biomicroscope (Nikon Corporation), to identify the presence of crystals or abnormalities in the cornea.

### In vivo confocal microscopy

Animals underwent in vivo CM scanning using a tandem scanning confocal microscope (TSCM; Tandem Scanning Corporation, Reston, VA) with a 24× surface-contact objective (numerical aperture, 0.6; working distance, 1.5 mm), encoder mike controller (Oriel 18011; Oriel, Stratford, CT) for focal plane control, and a camera (MTI VE-1000; Dage MTI, Michigan City, IN). One drop of preservative-free, Refresh Tears (Allergan, Irvine, CA) was placed on the tip of the objective as a coupling gel. All camera settings were kept constant throughout the experiment. For each eye, repeated data sets were obtained from select central and peripheral cornea locations. Three to five through-focus data sets were collected around these regions for assessment of cystine crystal volume using Metamorph Image Analysis software (Molecular Devices, Downingtown, PA).

### Ex vivo confocal microscopy

The eyes of sacrificed animals were enucleated and placed in 2% paraformaldehyde, phosphate buffered saline, pH 7.4, for 10 min, after which corneas were removed and fixed overnight. Tissue blocks (2×2 mm) were then obtained and stained en bloc overnight at 4 °C with nucleic acid stain (DAPI; Molecular Probes, Eugene, OR) to identify nuclei, phalloidin (Alexa Fluor Rhodamine phalloidin; Invitrogen, Carlsbad, CA) to identify cell cytoskeleton and CD45 anti-mouse FITC conjugated antibody (Abcam, Cambridge, MA) to identify inflammatory cell infiltration.

Samples were placed on a microscope (Axiovert 200; Zeiss, Jena, Germany) and imaged with a laser confocal microscope system (LSM 510 META; Zeiss) equipped with a mode-locked titanium/sapphire laser (Chameleon; Coherent, Santa Clara, CA). The fluorescent signal detection from CD45, rhodamine phalloidin, and nucleic acid stain (DAPI) was obtained using the 488-nm, 543-nm and 740-nm laser lines of the argon, and helium-neon, and infrared/femtosecond lasers.

### Quantitative assessment of cystine crystal content

To measure the cystine crystal content in the cornea, through focus image data sets were analyzed by Metamorph Image Processing Software (Molecular Devices, Downington, PA). Initially, the stromal regions were extracted from the through focus data set and then thresholded using the Threshold subroutine to include all high intensity pixels representing light scattering from the cystine crystals. Threshold regions were set to include pixels intensity from 100 to 255. Pixels within the threshold region were then counted using the “Measure” subroutine for all planes in the image stack to record the crystal volume. To calculate a “Percent Crystal Volume Index,” the crystal volume was divided by the extracted stromal volume.

### Statistical analysis

Each eye was considered independently and results are reported as the mean±standard deviation (SD). Differences between age groups were evaluated statistically using GraphPad Prism™ 3.0 (GraphPad Software Inc., San Diego, CA), considering one-way Anova and Newman-Keuls Multiple Comparison tests.

## Results

### Slit lamp photography

**Figure 1:**
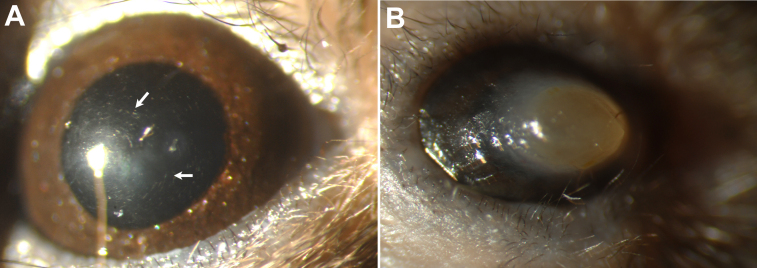
Slit lamp photography of a 7 month old *Ctns*^−/−^ mouse. **A**: The right eye with observed crystals in the cornea (arrows). **B**: The left eye of the same mouse with end stage corneal disease.

Small hyper-reflective needle-shaped deposits were first detected by biomicroscopy in the corneas of *Ctns*^−/−^ mice at 5 month of age, with more abundant crystals at 7 months (Figure 1A). Three eyes of the 14 *Ctns*^−/−^ animals developed phthisis bulbi, with central corneal opacification, loss of the normal ocular architecture and decreased ocular size at ages 7, 12, and 14 months (Figure 1B).

### In vivo confocal microscopy

**Figure 2:**
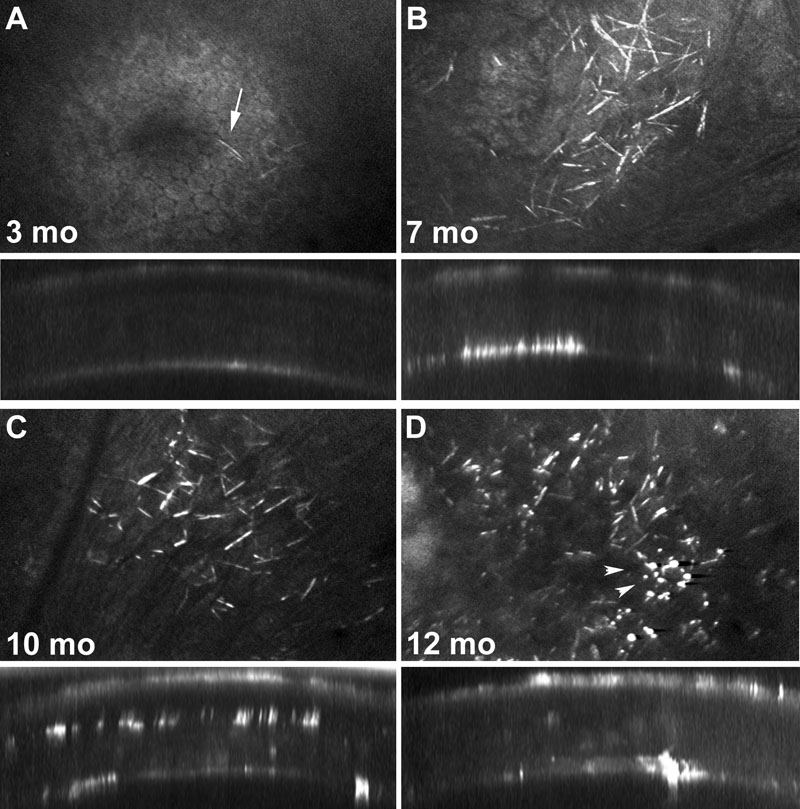
Confocal imaging time course. Confocal images of *Ctns*^−/−^ corneas at 3 months (**A**), 7 months (**B**), 10 months (**C**), and 12 months of age (**D**). Each panel shows a xy (upper) and xz (lower) slice through a 3-D stack. Cystine crystals were identified as small, 20 µm long, needle-like crystals in the peripheral and central cornea. Cystine crystals were first detected in 3 month old mice (**A**, arrow) and gradually increased in density with age up to 7 months (**B**). Older *Ctns*^−/−^ mice (10 and 12 month old) developed aggregates of brightly reflecting material, presumably cystine, within the central cornea (arrowheads; **C** and **D**, respectively).

Using in vivo CM, no corneal cystine crystals were noted in any of the C57Bl/6 control mice at any time point. In the *Ctns*^−/−^ mice, cystine crystals (identified as small, highly reflective needle shaped deposits) were first detected in the cornea at 3 months of age, located both centrally and peripherally in the posterior cornea near the endothelium (Figure 2A). By seven months of age, *Ctns*^−/−^ mice developed more abundant and larger needle shaped crystals in the mid posterior stroma, again noted in both the central and peripheral cornea (Figure 2B). At 10 month of age, crystals appeared thicker, shorter and were also noted in the anterior stroma (Figure 2C). By 12 month of age, deposits appeared more punctate with markedly fewer needle shaped crystals (Figure 2D). Overall, these morphological changes suggest that crystals increase in quantity until 7 month of age, after which they begin to form aggregates, without significant increase in crystal quantity.

### Measurement of cystine crystal content

**Figure 3:**
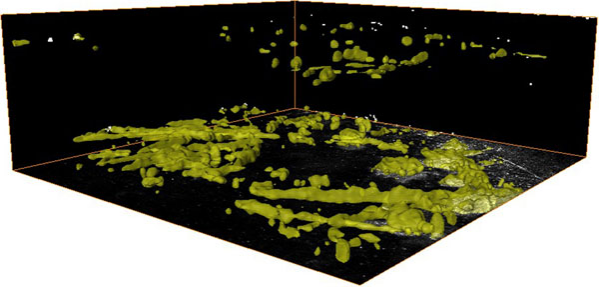
3-D distribution of crystals within the cornea of a 7month old *Ctns*^−/−^ mouse. Crystal volume was segmented from the 3-D in vivo CM scan by thresholding the images to contain just the crystal volume.

**Figure 4:**
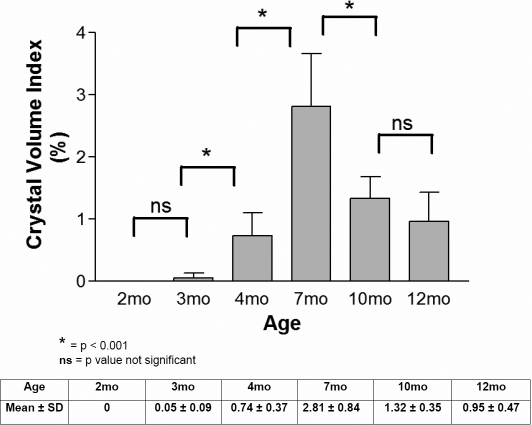
Cystine crystal quantification measured as the percentage (%) of crystals per stromal volume in different age groups. We observed a progressive increase in the crystal content in 3, 4, and 7 month old corneas that declined in the 10th and 12th month of age corneas (mo=month old; p<0.001 in 2 month versus 7 month, 2 month versus 10 month, 2 month versus 12 month, 2 month versus 4 month, 3 month versus 7 month, 3 month versus 10 month, 3 month versus 12 month, 3 month versus 4 month, 4 month versus 7 month, 4 month versus 10 month, 12 month versus 7 month, 10 month versus 7 month).

To quantify the progression of cystine crystal deposits in the cornea, we first segmented the crystal volume from the 3-D in vivo CM scan by thresholding the images to contain just the crystal volume. The data set was then volume rendered to show the 3-D distribution of crystals within the mouse cornea (Figure 3). 3-D renderings of the crystals were then used to calculate the crystal volume in each cornea, and the volume recorded as the percent of total stromal volume. Quantitation of crystal volume showed that at 2 months of age, no crystals were detected in any *Ctns*^−/−^ mice, however a small number of crystals were detected by 3 months of age. Crystal volume significantly increased 15-fold from 3 to 4 month of age and fourfold from 4 to 7 month of age (p<0.05) or 56 fold overall. From 7 to 10 month of age, there was a significant decrease in crystal volume to half that detected at 7 months. There was no significant change in crystal volume between 10 and 12 months of age (Figure 4).

### Ex vivo confocal microscopy

**Figure 5:**
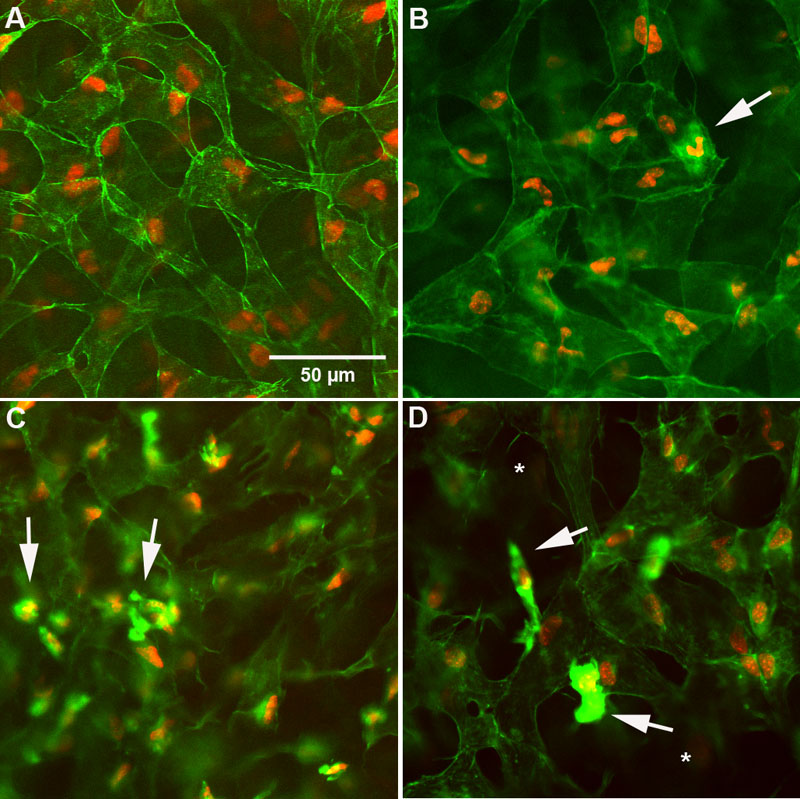
Corneal staining with actin and nuclei staining. **A**: Actin (Green) and nuclei (red) staining of cornea in normal C57Bl/6 mouse using confocal microscopy. We observed normal keratocytes cell bodies with dendritic morphology that stained in the periphery for actin. **B**: Actin and nuclei staining of corneas from *Ctns*^−/−^ mice at 5 months of age showing few inflammatory cells (arrow). Inflammatory cell infiltration appeared to increase in the 10th (**C**, arrows) and 12th (**D**, arrows) month of age with keratocyte dropout (asterisk).

**Figure 6:**
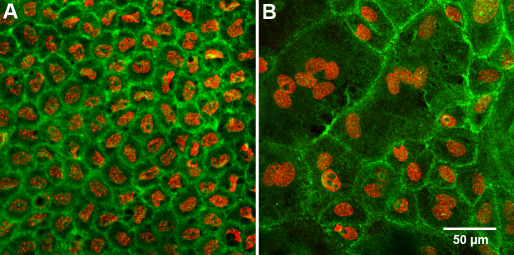
Actin (green) and Nuclei (red) staining of the endothelium in normal C57Bl/6 and *Ctns*^−/−^ mice. **A**: The endothelium of a 12 month old normal C57Bl/6 mouse, with the typical hexagonal shape and regular size. **B**: The endothelium of a 12 month old *Ctns*^−/−^ cornea. We observed an evident increased cell size with polymorphism, polymegathism, and multinucleation.

To evaluate changes to the keratocyte population associated with the deposition of cystine crystals, we stained corneas for actin to identify cell borders and DAPI to detect nuclei. Keratocytes in both the C57Bl/6 and 2–3 month old *Ctns*^−/−^ mice showed typical dendritic morphology and cortical actin staining (Figure 5A). Actin stained corneas of 5 month old *Ctns*^−/−^ mice demonstrated a few small cells near the limbus that stained intensively with phalloidin, suggesting the presence of stromal inflammation (Figure 5B). More abundant infiltrating cells were seen in the corneal periphery of 10 month old *Ctns*^−/−^ mice, often associated with disrupted keratocytes (Figure 5C). Both these features were more severe in 12 month old mice, with regions of keratocyte dropout (Figure 5C, asterisk) and decreased cell density (Figure 5D). Additionally, older mice (12 months) showed marked changes in the corneal endothelium, including loss of cell uniformity (polymegathism), polygonality (polymorphism) and increased cell size with formation of multinucleated cells (Figure 6).

**Figure 7:**
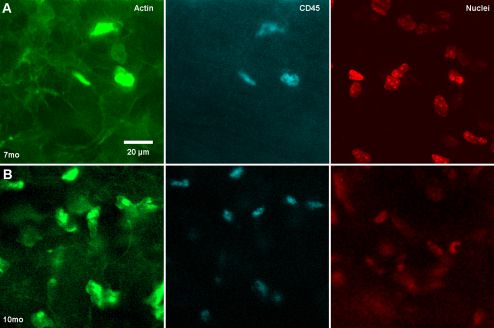
CD45, actin, and nuclei staining. CD45 (cyan), Actin (green) and Nuclei (red) staining of corneas from 7 (**A**) and 10 month old (**B**) *Ctns*^−/−^ mice. Panel **A** shows few CD45 positive stained cells in the limbus of 7 month old mice while panel **B** shows abundant CD45 stained cells in the limbus and central cornea of 10 month old *Ctns*^−/−^ mice.

To identify the presence of inflammatory cells, we stained corneas with antibodies to CD45, a marker for bone marrow derived cells. Cells with intense actin staining in the stroma showed positive staining for CD45 at 7 months of age (Figure 7A). Cells were located mostly in the periphery near the limbus but in older *Ctns*^−/−^ mice (10 months) inflammatory infiltrates were more prominent (Figure 6B) and present in both the peripheral and central cornea. Overall, these findings suggest that cystine crystal deposition leads to a chronic inflammatory response associated with loss of stromal keratocytes and endothelial cells.

## Discussion

Using in vivo and ex vivo confocal microscopy, we have characterized the clinical progression of corneal disease in the *Ctns*^−/−^ mouse. Our results show that cystine crystals are first noted at 3 months in the posterior cornea, later appearing in the anterior cornea and progressively increasing in density up to 7 months of age. More importantly, the volume of crystals appears to increase in quantity from 3 months to 7 month of age, after which they begin to form aggregates, without significant increases in crystal quantity. Increasing crystal volume also appeared to be associated with loss of keratocyte density and stromal inflammation from 5 to 10 months of age. Finally, many of the eyes appear to become phthisical as early as 7, but more commonly from 12 and 14 months of age.

Overall, our findings are similar to those of Kalatzis et al. [36], who characterized ocular cystinosis in the *Ctns*^−/−^ mouse by slit-lamp biomicroscopy, histology and measurement of tissue cystine levels. In this earlier report, corneal cystine crystals were first detected by slit-lamp biomicroscopy at 3 months of age, the same time that in vivo CM detects corneal crystals localized to the posterior cornea. Furthermore, quantitative measurement of tissue cystine levels showed a 62 fold increase in corneal cystine concentration from 3 months to 9 months of age. This was comparable to the 56 fold increase in corneal cystine crystal volume detected by in vivo CM in the current study. Taken together, these findings suggest that in vivo CM is as sensitive as the biochemical measurement of tissue cystine levels in characterizing the early progression of corneal cystinosis in the *Ctns*^−/−^ mouse. Importantly, in vivo CM is a non-invasive imaging technique that does not require sacrificing animals or the removal and processing of corneal tissue and therefore has distinct advantages to assessing corneal cystinosis by histology or biochemistry. Therefore, we propose that in vivo CM may be an objective and quantitative approach to assessing the progression of corneal cystinosis. More importantly, in vivo CM can be performed sequentially in the same eye over time, and can therefore be used as a sensitive approach to evaluating the effects of conventional and novel therapeutic strategies to inhibit or remove cystine crystals in the cornea. However, future studies are required to demonstrate this capability.

It should be noted that while the cystine levels were reported to remain stable after 9 months based on biochemical analyses in this earlier report, overall cystine crystal volume appeared to significantly decrease by in vivo CM. Since decreased crystal volume was associated with a morphologic change in crystal shape from needle-like to punctate, the apparent decrease may be due in part to differences in the strength of the reflected light signals from these two types of structures. Thus, aggregation of crystals to form punctate deposits may give a low signal, leading to a decrease in measured volume. Why crystals change in shape is not clear, however, the loss of keratocytes and the increased inflammatory cell infiltration suggests that as cells dies, crystals are broken up and perhaps phagocytosed by inflammatory cells. The fact that corneal tissue cystine levels as measured by Kalatzis et al. [36] remain stable after 9 months, also supports the possibility that as cells die released crystals dissolve into the extracellular matrix, or are phagocytosed and removed by wander cells.

Our observations of corneal crystal accumulation also support the appropriateness of the *Ctns*^−/−^ mouse as a model for human cystinosis, where 50%–70% of patients share the same genetic defect [5-8]. Although *Ctns*^−/−^ mice did not receive systemic treatment with oral cysteamine (as do humans), corneal disease has been shown not to be responsive to such systemic therapy. In cross-sectional human studies, corneal crystals developed between birth and 6 years of age, reaching a maximum by the mid teenage years [13,14,17,25,42]. This lack of corneal response to convential therapy underscores the importance of evaluating novel strategies to treat corneal cystinosis and the potential of the *Ctns*^−/−^ mouse model to identify efficacious therapies.

In human case studies, corneal crystal deposition has been reported to start in the periphery and proceed posteriorly and centripetally, with crystals seen in the entire corneal stroma by age twenty [43,44]. This is in contrast to our findings where corneal crystals were first noted in the posterior stroma, both centrally and peripherally but proceeded to the anterior corneal with time. Our in vivo confocal images did not detect any crystals in the corneal endothelium or epithelium, in contrast to human confocal microscopy reports of crystals being present in the epithelium but absent in the endothelium [40,41]. Additionally, our results showed changes in the corneal endothelium including cell loss and increased cell size with formation of multinucleated cells that has not been reported in any study in humans.

Thus, the *Ctns*^−/−^ mouse model seems to generally track with reported findings in humans. Finally, the findings of inflammatory cell migration, corneal neovascularization, and band keratopathy have also been reported in patients with nephropathic cystinosis who are in their twenties and thirties, again mirroring observations in the animal model [16].

In conclusion, the application of non-invasive CM, with the ability to sequentially evaluate and quantify crystal deposition in the same cornea, opens several interesting paths for future investigation. These include further studies assessing the effects of cystine crystals on keratocyte function and cell death. Such studies may help to elucidate molecular and cellular mechanisms involved in other organ systems such as the kidney and bone. In addition, in vivo CM of the *Ctns*^−/−^ mouse may be a sensitive and efficient screening tool for alternate therapies that until now have been hampered by the absence of an animal model. Such therapies might include novel drug delivery, gene transfer and stem cell transplantation, for instance, umbilical mesenchymal stem cell [45] and human corneal stromal stem cells [46] that had shown to be a promising treatment for congenital corneal diseases involving keratocyte dysfunction. Importantly, novel treatment strategies useful in the *Ctns*^−/−^ mouse may have wider application for other corneal diseases including mucopolysacchridoses, corneal dystrophy, and scarring.
